# *In vitro* α-amylase and hemoglobin glycation inhibitory potential of *Nigella sativa* essential oil, and molecular docking studies of its principal components

**DOI:** 10.3389/fphar.2022.1036129

**Published:** 2022-10-20

**Authors:** Mohammed Dalli, Nour Elhouda Daoudi, Farid Abrigach, Salah-eddine Azizi, Mohamed Bnouham, Bonglee Kim, Nadia Gseyra

**Affiliations:** ^1^ Laboratory of Bioresources, Biotechnology Ethnopharmacology and Health Faculty of Sciences, Mohammed First University, Oujda, Morocco; ^2^ Laboratory of Applied Chemistry and Environment, Faculty of Sciences, Mohammed First University, Oujda, Morocco; ^3^ Department of Pathology, College of Korean Medicine, Kyung Hee University, Seoul, South Korea; ^4^ Korean Medicine-Based Drug Repositioning Cancer Research Center, College of Korean Medicine, Kyung Hee University, Seoul, South Korea

**Keywords:** *Nigella sativa*, essential oil, antidiabetic, α-amylase, hemoglobin glycation

## Abstract

*Nigella sativa* is plant that is endowed with various pharmacological activities including antioxidant, anticancer, anti-inflammatory, antibacterial, antidiabetic, and immunostimulant. This study aims to investigate the antidiabetic activity of the *N. sativa* essential oil on two key enzymes the α-amylase and hemoglobin glycation. After the extraction procedure, the *N. sativa* essential oil, were subject to qualitative and semi-quantitative analysis using GC/MS, for the identification of the different bioactive compounds. This was followed by an evaluation of the *in vitro* inhibition capacity of the α-amylase and the hemoglobin glycation. Finally, a molecular docking study was conducted to determine the bioactive compounds responsible for the antidiabetic activity. The extracted essential oil showed the presence of different bioactive compounds including α-phellandrene (29.6%), β-cymene (23.8%), 4-caranol (9.7%), thymol (7%). The *N. sativa* essential oil was found to be endowed with an antiradical scavenging activity with an IC_50_ of (7.81 ± 0.08 mg/ml), and to have a ferric reducing activity with an IC_50_ value of (7.53 ± 0.11 mg/ml). The IC_50_ value for the α-amylase inhibitory activity was 0.809 mg/ml, indicating an inhibitory impact of the enzyme. The IC_50_ value for the *N. sativa* essential oil’s hemoglobin antiglycation activity was 0.093 mg/ml. For most predominating phytochemicals present in the *N. sativa* essential oil, molecular docking studies against human pancreatic α-amylase and human hemoglobin enzymes revealed that these compounds can serve as lead molecules to develop new antidiabetic compounds.

## 1 Introduction

Chronic hyperglycemia is a symptom of diabetes, which is a metabolic condition. (Raffel et al., 2014). According to the WHO, diabetes is among the top 10 causes of death in 2019. If the glucose elevation is left untreated could lead to severe consequences such as microvascular (coronary disorder, arterial disease, and strokes) and microvascular (neuropathy, retinopathy, and nephropathy) alterations (Bhaskarachary and Joshi, 2018), (Gulçin et al., 2018). The diabetes disorder is in continuous increase and is estimated that by the year 2045 the number of diabetic adults will be about 700.2 million (Dandekar et al., 2021). There are mainly two types of diabetes type 1 which is related to pancreatic Beta cells impairment which causes a deficiency in insulin secretion while type 2 that is related to insulin resistance (Bahmani et al., 2014), (Taslimi and Gulçin, 2017). The gastrointestinal tract is an environment that is very known for its richness with different enzymes such as α-amylase which plays a principal role in splitting the starch into maltose and glucose (Smith and Morton, 2010). The circulating glucose in the case of chronic hyperglycemia can be linked to proteins (glycation) (Lima and Baynes, 2013), (Golshahi and Bahramikia, 2020), which has severe consequences such as the change in the proteins functioning, high aggregation, modification in absorbance peak, especially within the lens which is responsible for the opacification and cataracts in diabetic subjects. Also, the glycation could play a potential role in some disorders related to age such as Alzheimer and brain damage, in addition to that the glucose linkage to DNA could cause a cell dysfunction (Lee et al., 2007).

*N. sativa* (NS) or black cumin is a plant native to the Mediterranean region (Malhotra, 2012). This plant, which is a member of the Ranunculaceae family, has been utilized in traditional medicine as a body energy stimulant that also helps people feel less stressed and recover from fatigue more quickly (Amin and Hosseinzadeh, 2016). In Morocco black cumin is also used in traditional medicine for respiratory problems (Bellakhdar et al., 1991), as an anticancer agent when mixed with honey (Kabbaj et al., 2012), this panacea is also used to treat hypertension, stomachache, and rheumatic pain (Fakchich and Elachouri, 2021). Several research have recently revealed that NS is a plant rich in bioactive substances like as phenolic acids, flavonoids, and alkaloids. On the other hand, this plant was exhaustively studied for its different health benefits (Dalli et al., 2022), where the plant was found to have immunomodulatory, anti-inflammatory (Majdalawieh and Fayyad, 2015), antimicrobial (Deloer et al., 2019; Dalli et al., 2022), antiviral (Esharkawy et al., 2022), and antidiabetic activity (Tiji et al., 2021), (Dalli et al., 2021b).

The current study aims to evaluate the antidiabetic activity of *N. sativa* essential oil against the pancreatic α-amylase and the hemoglobin glycation. The study was accompanied by *in silico* molecular docking studies against these two targets to determine computationally the putative bioactive compounds responsible for the antidiabetic potential of *N. sativa* essential oil extract.

## 2 Materials and methods

### 2.1 Vegetal matter

We purchased the *N. sativa* seeds from a herbalist. Under the code HUMPOM471, a plant sample was placed into the faculty herbarium.

### 2.2 Extraction of NS essential oil

The *N. sativa* seeds were placed in a dark area and left there until they dried naturally. The dried seeds were taken and turned into fine powder. Then, 100 g were taken and put into a round-bottom flask with 300 ml of distilled water. The mixture was subject to hydrodistillation using a Clevenger apparatus. After the extraction (2 h 30 min), the essential oil was recovered and stored in a dark glass container for further use.

### 2.3 *N. sativa* essential oil analysis using GC-MS

The analysis of the essential oil from hydrodistillation was performed in a Shimadzu GC-2010 gas chromatography (GC) that is equipped with a column (30 m × 0.25 mm, 0.25 μm film thickness, 5% phenyl methyl siloxane). The gas chromatography is coupled to GC-MS-QP2010 mass detector. The helium carrier gas is attached to the gas chromatography with a steady pressure (100 KPa). The oven of the GC was put on 50°C for about 1 min. Then, the T was increased with a rate of 10°C per minute to reach a temperature of 250°C. A 1 µl solution of the essential oil already prepared in n-hexane and injected. The GC-MS system was in scan mode when used. By comparing the constituents’ mass spectra to information stored in the National Institute of Standards and Technology’s computer library (NIST147), the constituents’ identities were ascertained.

Data was gathered and handled utilizing LabSolutions (version 2.5).

### 2.4 Antioxidant potential of NS Eo

#### 2.4.1 Antiradical scavenging activity against DPPH

The antiradical scavenging potential of NS extracted Eo was investigated with the method proposed by Manzocco et al. (1998) (Manzocco et al., 1998). From each concentration an aliquot of 200 µl was combined with 1.8 ml of DPPH (0.5 mM) already prepared in methanol. The incubation of the reaction mixture was assessed under dark conditions for a duration of 30 min at room temperature. Finally, the reaction mixture’s absorbance was compared to a blank at 517 nm. All measurements were carried out three times. A positive control was employed, which was ascorbic acid.

The percentage of radical scavenging activity was calculated according to the formula:

$$\%\;radical\;scavenging\;activity=\frac{\left({Abs}_{Control}-{Abs}_{Sample}\right)}{{Abs}_{Control}}\times 100$$ (1)

#### 2.4.2 Ferric reducing power assay

The ferric reducing power assay was evaluated following the protocol elaborated by (Dehpour et al., 2009) with some modifications. Different concentrations of NS essential oil were prepared. Then, an aliquot of 0.5 ml was taken from each concentration and added to a solution formed by 1.25 ml of potassium ferricyanide (K_3_Fe (CN)_6_) and 1.25 ml of phosphate buffer solution (PBS) (0.2 mM, pH 6.6). Afterward, The resulting mixture was given a 20-min incubation period in a water bath at 50°C, 1.25 ml of trichloroacetic acid (TCA) was added after cooling to stop the reaction. For 10 min, the various tubes were centrifuged at 3,000 rpm. From each tube, 1.25 ml was taken and mixed with 0.25 ml of ferric chloride (FeCl3). Finally, the absorbance was measured at 700 nm against the blank. The positive control used in our study was the ascorbic acid.

### 2.5 *In vitro* inhibition of α-amylase

The evaluation of the capacity of the NS Eo to inhibit the action of the α-amylase enzyme was measured following the protocol proposed by Thalapaneni et al. (2008). 200 µl of the pancreatic -amylase enzyme and 200 µl of PBS (pH = 6.9) were combined with 200 µl of each prepared concentration of Eo or acarbose. A preincubated of the mixture for 10 min at 37°C was done. Each tube received an aliquot of 200 µl of a prepared starch (1 percent) solution, which was then incubated at 37°C for 20 min. The process was then stopped by adding 600 µl of DNSA to the mixture. Subsequently, the different tubes were incubated at a high temperature (100°C) for about 8 min then directly cooled in an ice bath. At the end, the tubes (EO and acarbose) were diluted by adding a 1 ml of distilled H_2_O. At 540 nm, the absorbance was then measured.

The formula below was used to compute the percentage of α-amylase enzyme inhibition:

$$Inhibition\;percentage\;\left(\%\right)=\frac{\left(ODtest-ODcontrol\right)}{ODtest}\times 100$$ (2)

### 2.6 *In vitro* hemoglobin antiglycation activity

In order to evaluate the *N. sativa* essential oil hemoglobin antiglycation effect, the method described by Chauhan et al. (2019) was adopted. An aliquot of 1 ml hemoglobin 5% solution was taken and added to 5 µl of gentamicin, 25 µl of the NS essential oil or the gallic acid at different concentrations, and finally the PBS (pH = 7.4). Afterward, an aliquot of glucose (1 ml) (4 mg/ml) was included in order to prompt the reaction.

The reaction mixture was then kept at room temperature for 72 h while being kept in a dark area. At 443 nm, the mixture’s absorbance was measured.

The following formula was used to calculate the inhibition percentage:

$$Inhibition\;percentage\;\left(\%\right)=\frac{\left(ODcontrol-ODblank\right)-\left(ODsample-ODsampleblank\right)}{ODcontrol-OD\;control\;blank}\times 100$$ (3)

### 2.7 Computational study

#### 2.7.1 Compounds preparation

Based on the GC-MS analysis, the most dominant compounds identified in the essential oil of NS seeds (>6%) were chosen for molecular docking calculations. 3D structures as SDF format of following selected biocompounds were retrieved from PubChem database (https://pubchem.ncbi.nlm.nih.gov/): α-Phellandrene (CID: 7460), β-Cymene (CID: 10812), 4-Caranol (CID: 86056), Thymol (CID: 6989), α-Pinene (CID: 6654), β-Pinene (CID: 14896). Acarbose (CID: 41774) and Gallic acid (CID: 370) were used as standard drugs.

#### 2.7.2 Preparation of the target protein

The crystal structures of the α-amylase from the human pancreas in complex with nitrite and acarbose (PDB code: 2qv4) at 1.97 Å resolution (Maurus et al., 2008) and the crystal structure of deoxy human hemoglobin complexed with two L35 molecules (PDB code: 2d60) at 1.70 Å resolution (Yokoyama et al., 2006) were used as enzymes in this study, and their structures were obtained from the RCSB Protein Data Bank (www.rcsb.org). The target receptors were prepared before docking by deleting all water molecules, inhibitors, co-crystallized molecules, and adding missing hydrogen atoms (polar only) with the aid of BIOVIA Discovery Studio Visualizer 2020.

#### 2.7.3 Molecular docking study

To investigate the binding method of the selected compounds of NS essential oil with α-amylase (αA) enzyme and human hemoglobin (HbA) protein, molecular modeling studies were carried out using virtual screening software PyRx 8.0 (Dallakyan and Olson, 2015). Briefly, under PyRx, all ligand structures were optimized before docking with the target protein. The grid box dimensions were fitted to enclose the active site pocket for each receptor. The grid box for αA was 30 × 30 × 30 grid points in size centred at 12.83 × 47.14 × 26.59; a grid map of 40 × 40 × 40 grid point in size centred at 14.18 × 02.51 × 11.49 was created for HbA. Gibb’s binding free energy (ΔG) was calculated and explored to determine the binding affinity for each ligand for the target receptor. For the best poses of ligands and standard drugs, the interaction details in the 2D format of ligand-receptor complexes were generated and visualized with BIOVIA Discovery Studio 2020.

### 2.8 Statistical analysis

ANOVA was used to do statistical analysis on the data, which included multiple-group comparisons (One-way-analysis of variance). The unpaired T student was used to compare the IC_50_ of the sample and the drug.

## 3 Results and discussion

### 3.1 NS Eo identified bioactive compounds

**FIGURE 1:**
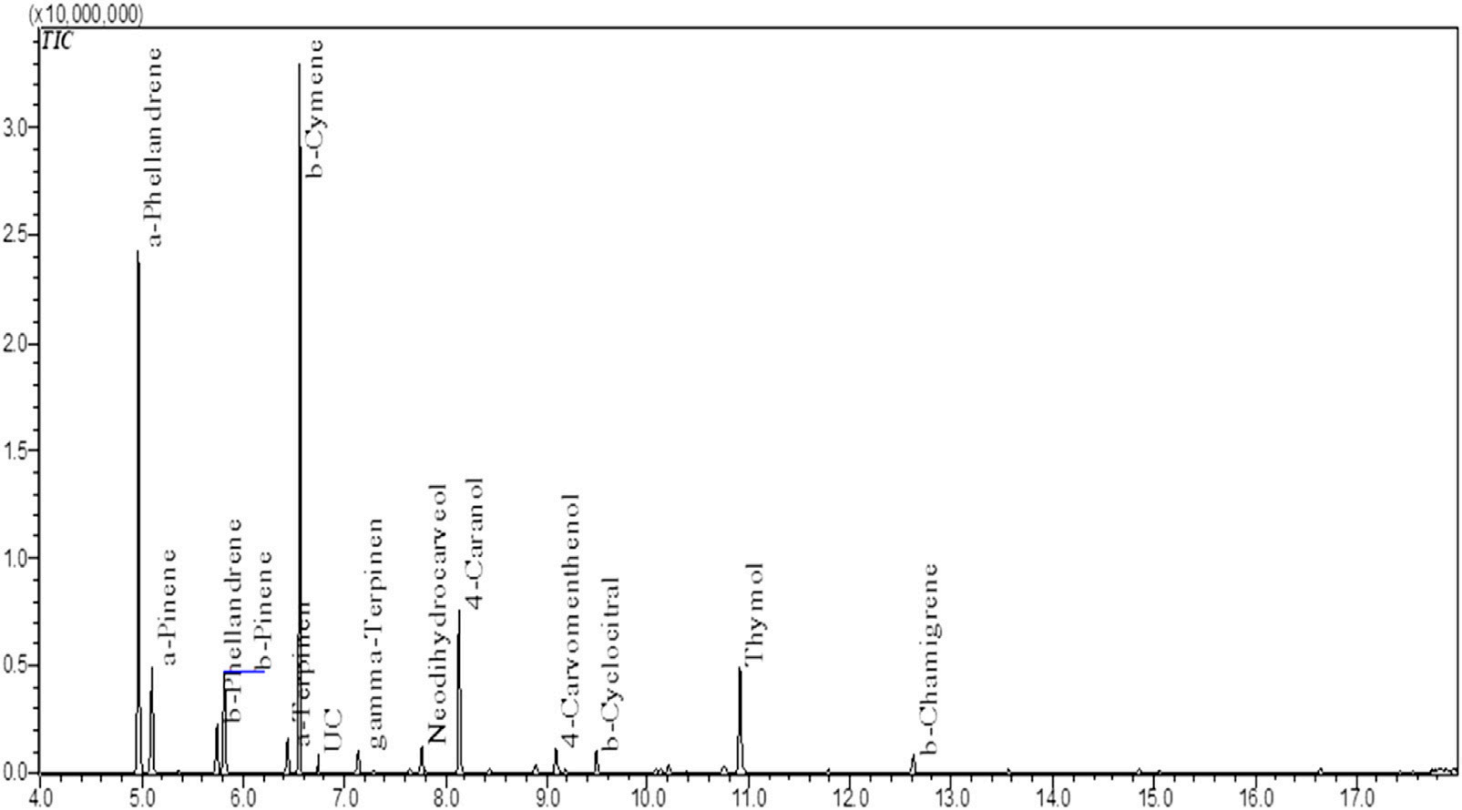
Total Ion Chromatogram (TIC) of NS essential oil by GC-MS (Dalli, 2021).

The NS Eo was extracted using hydrodistillation with a modified-Clevenger apparatus, yielding 1.16 percent. The chemical compounds of the essential oil were examined using a gas chromatography linked to mass spectrometry. The components’ identities were discovered through a comparison of their mass spectra with data held at the (NIST). The chemical composition of the Eo was performed in a previous study. The results obtained are drafted in Figure 1; Table 1. The monoterpenes constituted the highest percentage among all the identified compounds. The compound α-phellandrene has the largest amount of 29.64 percent, followed by β-cymene with 23.82 percent, 4-caranol with 9.77 percent, and thymol with 7%, while the other compounds have a proportion of 6 percent or less.

**TABLE 1:** The identified chemical components in NS essential oil by GC-MS. (Dalli, 2021).

Compound	RT (min)	%	Compound	RT (min)	%
α-Phellandrene	**4.97**	**29.64**	Cuminal	10.08	0.13
α-Pinene	**5.10**	**6.29**	Carvone	10.14	0.15
UC	5.27	0.13	Thymoquinon	10.21	0.50
Camphene	5.36	0.12	Cis-Verbenol	10.27	0.05
UC	5.44	0.11	4-Carene	10.38	0.09
β-Phellandrene	5.74	2.87	Bornyl acetate	10.76	0.59
β-Pinene	**5.82**	**6.22**	**Thymol**	**10.92**	**7.00**
β-Myrcene	5.98	0.15	UC	11.14	0.04
Pseudocumol	6.07	0.32	β-Terpinyl acetate	11.65	0.02
UC	6.18	0.05	α-Longipinene	11.78	0.18
α-Thujene	6.24	0.11	Columbin	12.11	0.02
UC	6.35	0.20	UC	12.31	0.01
α-Terpinen	6.44	2.25	UC	12.41	0.04
β-Cymene	**6.56**	**23.82**	β-Chamigrene	12.62	1.14
Thujol	6.98	0.02	α-Bisabolene	12.77	0.03
ɤ-Terpinene	7.14	1.25	UC	13.00	0.01
cis-β-Terpineol	7.29	0.11	UC	13.12	0.01
Terpinolen	7.64	0.15	UC	13.28	0.02
Neodihydrocarveol	7.77	1.63	UC	13.49	0.03
UC	8.04	0.04	2-Tridecanone	13.57	0.07
4-Caranol	**8.14**	**9.77**	UC	13.64	0.02
UC	8.29	0.03	β-Bisabolene	13.83	0.03
UC	8.45	0.25	UC	13.99	0.02
UC	8.57	0.01	Isoledene	14.15	0.01
Camphor	8.62	0.05	UC	14.40	0.02
β-Cyclocitral	8.75	0.03	UC	14.72	0.02
Limonene epoxide	8.89	0.52	UC	14.85	0.07
UC	8.99	0.03	UC	15.06	0.06
4-Carvomenthenol	9.10	1.44	UC	15.17	0.05
UC	9.19	0.15	UC	15.62	0.01
UC	9.25	0.01	UC	15.87	0.05
UC	9.30	0.03	UC	16.04	0.02
UC	9.40	0.07	Globulol	16.15	0.03
β-Cyclocitral	9.50	1.39	UC	16.64	0.06
UC	9.55	0.02	UC	16.88	0.02
UC	9.74	0.08			

Abbreviations: RT, Retention Time, UC, Unknown Compound.

The bold values are of the dominant compounds in our essential oil

### 3.2 Antioxidant activity

The NS essential oil extracted by hydrodistillation has shown an antiradical scavenging activity with an IC_50_ of about 7.809 mg/ml. While it was also found that the NS essential oil is endowed with a ferric reducing power with an IC_50_ of 7.531 mg/ml. A highly significant difference was observed when compared to ascorbic acid and this was for both antiradical scavenging activity against DPPH, and the ferric reducing power assay (*p* < 0.0001) (Table 2). Our essential oil was less active when compared to the essential oil in Burits and Bucar. (2000) study with an IC_50_ of 460 μg/ml. In the same study, it was mentioned that carvacrol and thymoquinone were endowed with high antioxidant activity. It was also reported that the α-pinene a compound present in our Eo with a percentage of 6.29% was found to be endowed with a strong antiradical and ferric reducing power activity (Wang et al., 2019). On the other hand, the thymol (7%) was mentioned to have a high antioxidant capacity at a very low concentration (Nagoor Meeran et al., 2017). Finally, the β-cymene a compound present in our Eo (23.82%) was reported to have strong antioxidant activity. It was also noted that this compound showed a significant reduction of lipid peroxidation and an elevation of superoxide dismutase (SOD) and catalase (CAT) (De Oliveira et al., 2015).

**TABLE 2:** *In vitro* antioxydant potential of *N. sativa* essential oil using DPPH a,d FRAP assays.

	Antiradical scavenging assay IC_50_ mg/ml	Ferric reducing power assay IC_50_ mg/ml
Essential oil	7.81 ± 0,08****	7.53 ± 0.11****
Ascorbic acid	0.023 ± 0.003	0,0627 ± 0,01

### 3.3 α-amylase inhibition (*in vitro)*

**FIGURE 2:**
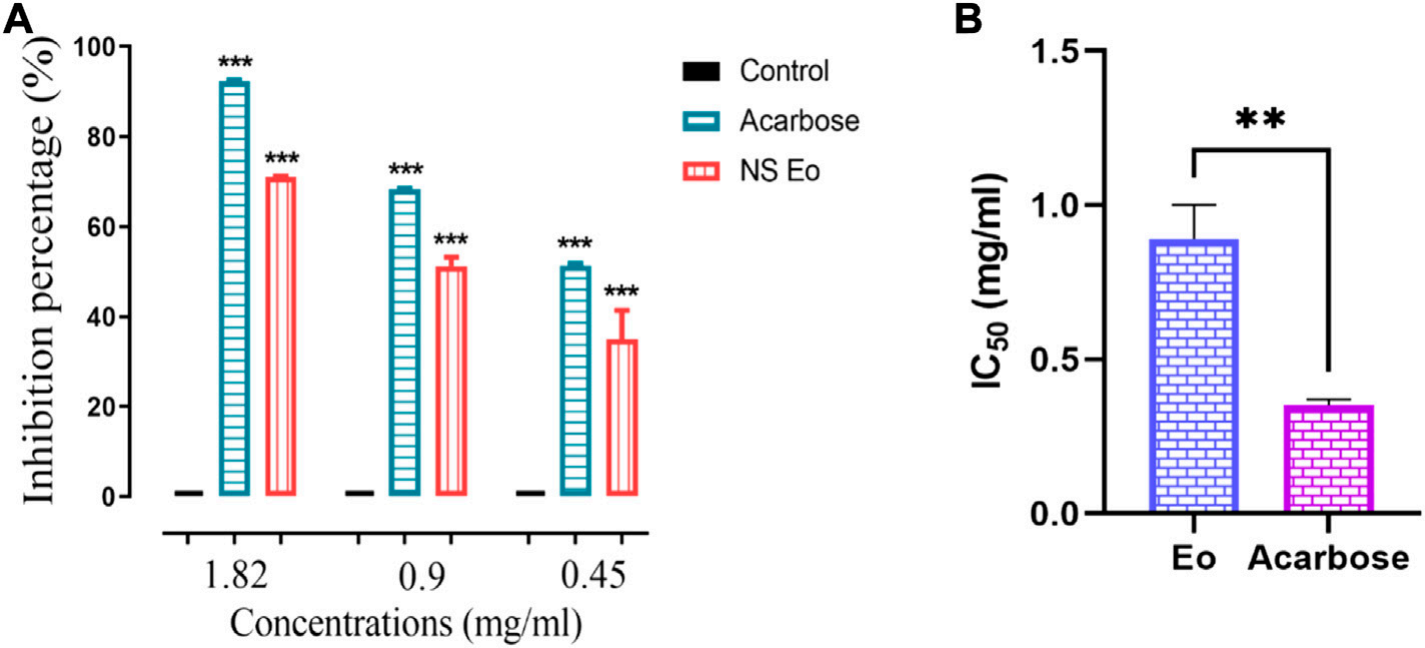
**(A)** NS essential oil and acarbose against pancreatic α-amylase, **(B)** IC_50_ of the essential oil and acarbose. (*n* = 3) ***p < 0.001 as compared with the control. (*n* = 3). **p < 0.001 when comparing the IC_50_.

The above-mentioned technique was used to determine the NS essential oil inhibitory potential. The essential oil obtained using hydrodistillation was found to be a very potent inhibitor of the pancreatic α-amylase in a dose-dependent manner (Figure 2). At a high concentration of 1.82 mg/ml, the essential oil had an inhibition percentage of 71.07%, while the acarbose at the same concentration had the highest inhibition percentage with a value of 92.24% (*p* < 0.0001). The IC_50_ value recorded for the black cumin essential oil was about 0.809 ± 0.11 mg/ml. Acarbose used as positive control showed a lower IC_50_ value equal to 0.35 ± 0.02 mg/ml. The statistical analysis performed showed the existence of highly significant difference between the IC_50_ of the acarbose used as positive control and the IC_50_ of the NS essential oil (*p* < 0.001). Our essential oil performed a moderate activity when comparing the IC_50_ recorded in our Essential oil that was higher than that found when testing the volatile compounds from *Sabina chinensis* (IC_50_ 187.08 μg/ml), and from *Eruca vesicaria* (Stems, fruits, roots, leaves) (Gu et al., 2018), (Hichri et al., 2019). Also, the essential oil extracted from *Cedrus libani* has showed a high inhibition potential of α-amylase with an IC_50_ value of 0.14 mg/ml (Loizzo et al., 2007). Sahin et al. (Sahin Basak and Candan, 2010) mentioned that the p-cymene, and α-pinene two monoterpenes endowed with an inhibitory activity of α-amylase with an IC_50_ of 36.5 μL/ml and 32.22 μl/ml respectively (Sahin Basak and Candan, 2010). Also the β-pinene was noted to have an inhibitory potential on α-amylase (Jelenkovi et al., 2014).

### 3.4 *In vitro* hemoglobin antiglycation activity

**FIGURE 3:**
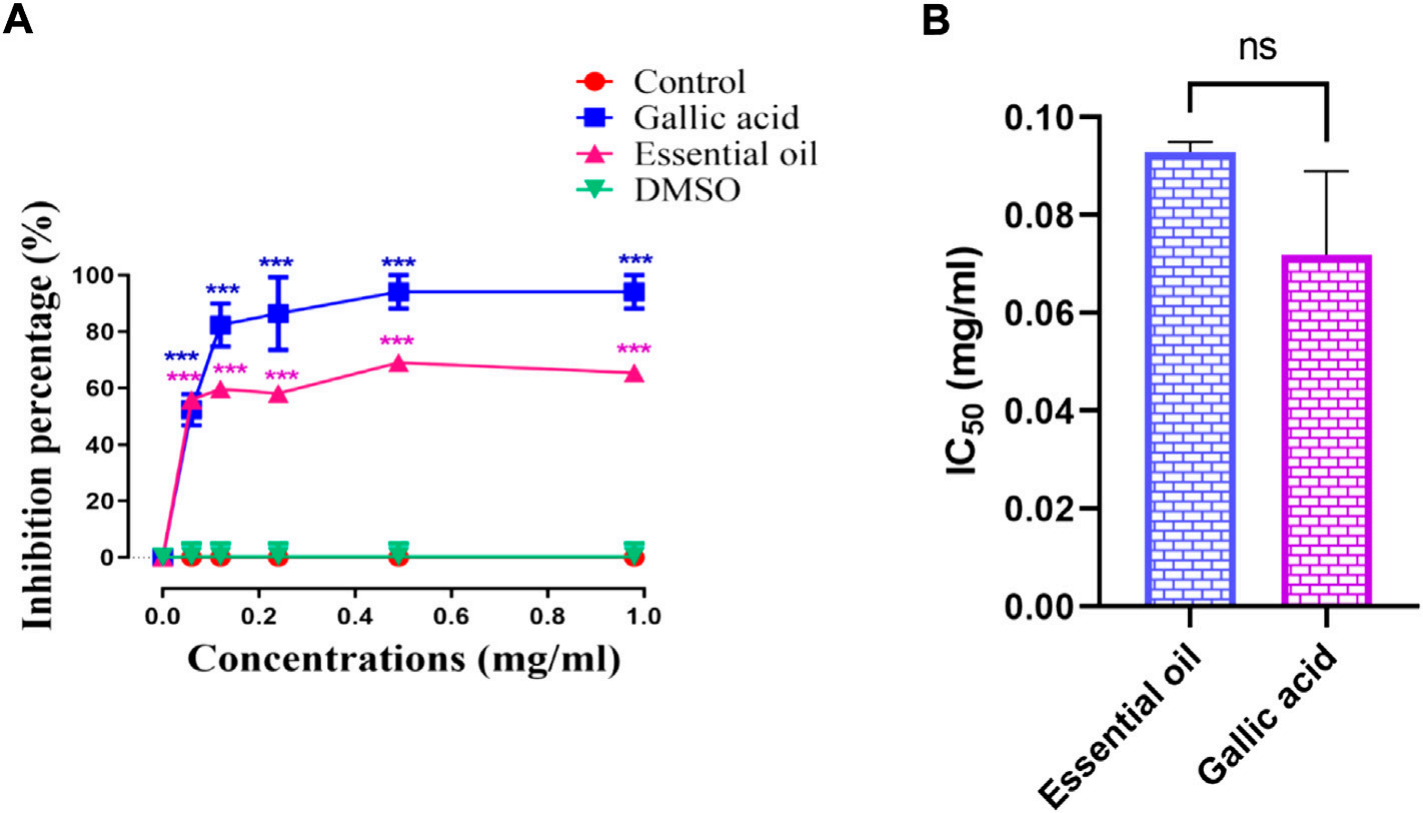
**(A)** Antiglycation activity of NS essential oil and gallic acid, **(B)** IC_50_ of NS essential oil and gallic acid. (*n* = 3). ***p < 0.001 as compared with the control. (*n* = 3). NS *p* > 0.05 when comparing the IC_50_.

The NS essential oil antiglycation effect was conducted *in vitro* using the hemoglobin antiglycation method. The results obtained indicate that the inhibition of the hemoglobin glycation was in a dose-dependent manner (Figure 3). At a concentration of 1 mg/ml of NS volatile compounds, it was observed an inhibition of 65.42% while the gallic acid gave an inhibition percentage of about 94.08% at the same concentration (*p* > 0.05). The IC_50_ value registered was 0.072 ± 0.017 mg/ml for gallic acid and 0.093 ± 0.002 mg/ml for NS essential oil (*p* > 0.05). On the other hand, different studies assessed on the essential oil of *Juniperus oblonga* and *Juniperus sabina* branches were able to inhibit hemoglobin glycation in dose dependent manner but using high concentrations (200, 400, and 600 μg/ml) (Emami et al., 2012).

### 3.5 Molecular modeling studies

**FIGURE 4:**
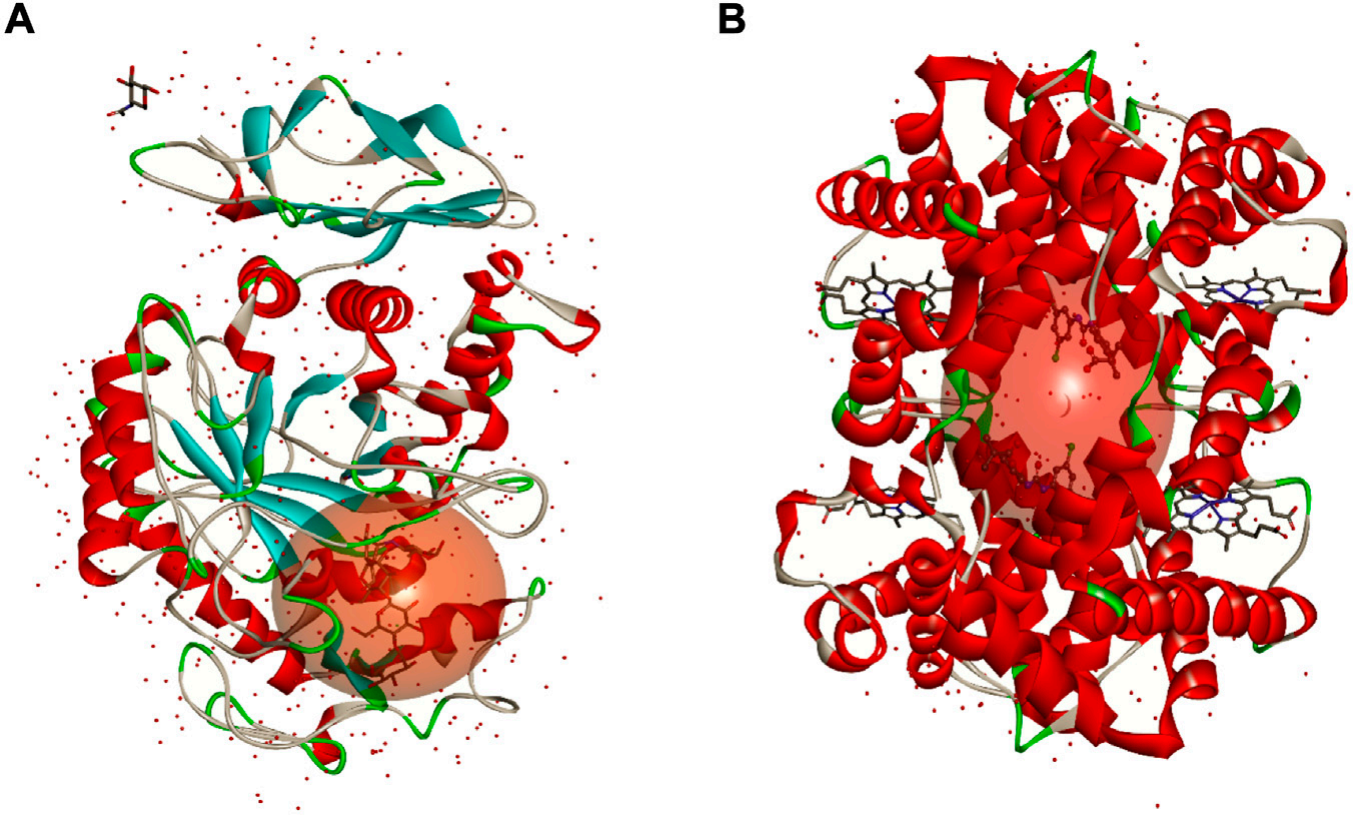
3D structures of α-amylase **(A)** and human haemoglobin **(B)**. The sphere in red represents the active site of each protein.

The knowledge of the binding mode and the probable binding position of a given drug within the protein scaffolds plays a vital role to determine and understand the effectiveness of the molecule to serve as a therapeutic agent (Paul et al., 2014). In this context, molecular docking is one of the powerful computational tools used to predict and identify the possible binding mode that occurs between a molecule and a given target protein or receptor (Abrigach et al., 2018). In this work, molecular docking studies were accomplished in order to establish to determine which chemicals are best suited to be the most abundant compounds in NS essential oil, with α-amylase (αA) and human hemoglobin (HbA) (Figure 4). The compounds with the lowest binding energy (ΔG) with the protein indicate the highest inhibitory potential.

### 3.6 Molecular docking with human pancreatic α-amylase receptor

**FIGURE 5:**
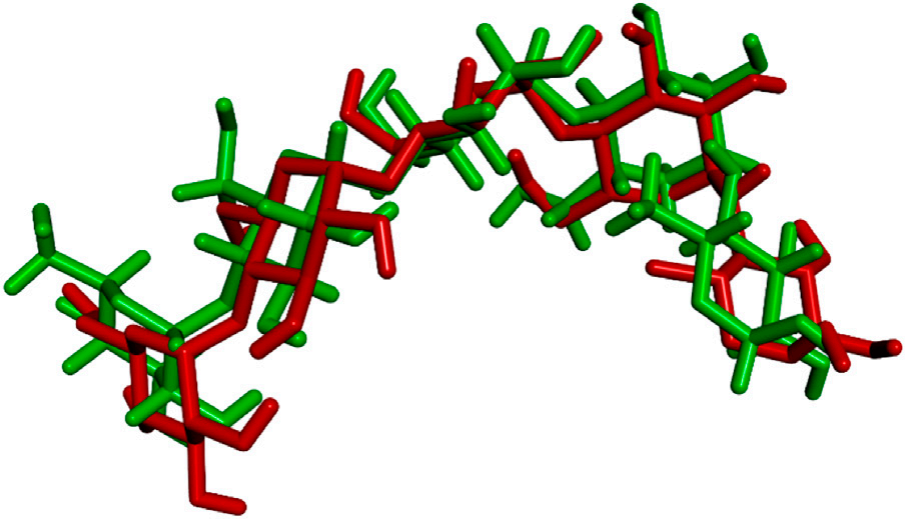
Superposition of the origin acarbose derivative (green) and the best re-docked acarbose derivative pose (red) in the binding pocked of human pancreatic α-amylase protein (2qv4). RMSD value equal to 1.298 Å.

The docking procedure was initiated by re-docking the native ligand (acarbose derivative), to the binding site of the crystal structure of the human pancreatic α-amylase protein (2qv4) for validating the docking protocol. In the crystal structure of α-amylase, the co-crystallized ligand showed conventional hydrogen bonds with Thr163, Ala106, Gly164, Val107, Gln63, Tyr62, His299, Arg195, Asp300, and Glu233 amino acids. Moreover, this ligand also interacts with several other residues of protein such as Ile51, Asn105, Gly104, Leu165, Glu60, His101, Leu162, Tyr151, Ile235, Lys200, His201, Asp197, Ala198, Asn298, Trp58, Trp59, and His305 *via* Van der Waals forces. According to the obtained results, the re-docked acarbose derivative showed similar conformational poses and was found to have almost interactions with the similar amino acids where the co-crystallized acarbose interacted. The RMSD value which gives the average deviation between the crystal structure pose and the re-docked pose of the acarbose derivative was 1.298 Å (Figure 5). This value is significantly lower than the maximum allowable value of 2.0 Å, which confirms the validity of the docking method. The binding energy of the best-docked acarbose derivative pose was −9.0 kcal/mol.

Analysis of the docking outcomes summarized in Table 3, indicated that acarbose, a standard type 2 antidiabetic used as a standard drug, displayed high binding energy of −8.2 kcal/mol. The binding potential of the six abundant phytochemicals found in the Eo was shown to be between −6.0 and −5.5 kcal/mol, with -6.0 kcal/mol being the lowest binding energy for thymol and hence the highest α-amylase inhibition. 4-caranol, α-phellandrene, and β-cymene also showed high binding potential. α-pinene and β-pinene exhibited moderate α-amylase inhibitory potential. These values were found to be comparable with acarbose, which suggests that our essential oil is rich in compounds that, alone or synergistically, lead to decreased α-amylase activity, as observed in the *in vitro* α-amylase inhibition assay.

**TABLE 3:** Docking outcomes of major compounds in the NS essential oil against the human pancreatic α-amylase protein.

Compound	Binding energy (kcal/mol)	Involved receptor residues	Type of interactions
α-Phellandrene	−5.9	Tyr62, His299, Asp197, Asp300, Arg195, Trp58, Trp59, Leu165	Pi-Sigma, Pi-Pi stacked, VDW
β-Cymene	−5.8	Tyr62, His299, Trp58, Asp197, Asp300, Ala198, His101, Trp59, Leu165	Pi-Sigma, Pi-Alkyl, Pi-Pi stacked, VDW
4-Caranol	−5.9	Asp197, Tyr62, His299, Trp58, Trp59, Asp300, Arg195, Ala198, His101, Leu165	HB, Pi-Sigma, Pi-Alkyl, VDW
Thymol	−6.0	Tyr62, His299, Asp197, Trp58, Trp59, Asp300, Arg195, His101, Leu165	Pi-Sigma, Pi-Alkyl, Pi-Pi stacked, VDW
α-Pinene	−5.6	Tyr62, His299, Trp58, Trp59, Asp300, His101, Leu165	Pi-Sigma, Pi-Alkyl, VDW
β-Pinene	−5.5	Tyr62, His299, Trp58, Trp59, Asp300, Asp197, His101, Leu165	Pi-Sigma, Pi-Alkyl, VDW
Acarbose	−8.2	Asp300, Arg195, Glu233, Asp353, Asp356, His305, Thr163, Tyr62, His101, His299, Ile235, Asn352, Asp197, Ala198, Leu162, Leu165, Trp58, Trp59, Trp357, Val98, Val354, Ile235	HB, VDW

Abbreviation: HB, Hydrogen Bond; VDW, Van der Waals forces.

**FIGURE 6:**
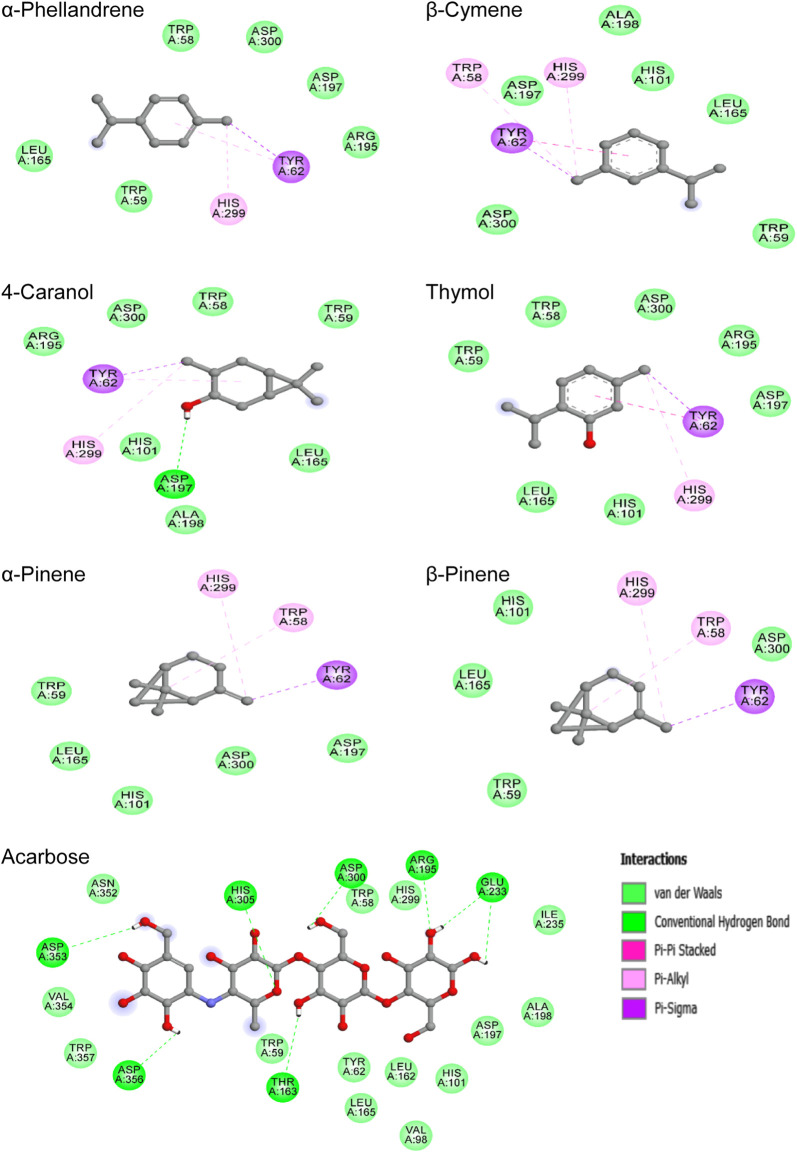
2D-representations of the best-docked poses of the studied ligands in the active pocked of human pancreatic α-amylase.

The chemical bonding mode of the complexes formed between studied compounds and the binding pocket residues of α-amylase are displayed in Figure 6. Acarbose binds in the active site of protein *via* hydrogen bonds with seven amino acids, namely, Asp300, Arg195, Glu233, Asp353, Asp356, His305, and Thr163; and also showed hydrophobic interactions (VDW) with Tyr62, His101, His299, Ile235, Asn352, Asp197, Ala198, Leu162, Leu165, Trp58, Trp59, Trp357, Val98, Val354, and Ile235. For all abundant phytoconstituents, predominantly hydrophobic interactions were responsible for the binding of these molecules to the active site of α-amylase. Also, it was observed that these molecules formed interactions with similar residues with slight differences. The most stable complex (−6.0 kcal/mol) thymol—α-amylase was stabilized by one Pi-Sigma, one Pi-Pi stacked, one Pi-Alkyl, and seven VDW interactions involving the amino acids Tyr62, His299, Asp197, Trp58, Trp59, Asp300, Arg195, His101, and Leu165. For the other complexes, analysis of the best-docked poses revealed that they mainly exhibited numerous hydrophobic interactions including Pi-Sigma, Pi-Alkyl, Pi-Pi stacked, and VDW, except 4-Caranol which in addition to these interactions form a hydrogen bond with Asp197 residue.

On the other hand, the study of the X-ray crystal structure of human pancreatic α-amylase demonstrated the presence of three characteristic amino acids namely Asp197, Asp300, and Glu233 which contribute to the overall catalytic action in starch-hydrolyzing enzyme (Kumar et al., 2017; Anigboro et al., 2021; Şahin et al., 2022). Asp197 was reported to act as a catalytic nucleophile during starch hydrolysis, Glu233 amino acid plays the role of an acid-base catalyst, whereas Asp300 is used for optimizing the orientation of the substrate (Alqahtani et al., 2019; Kikiowo et al., 2020).

Interestingly, acarbose was found to form strong hydrogen bonds with all these three enzyme key residues, which may explain its lower binding potential value in comparison with other studied compounds. Meanwhile, the docking data also showed that all the selected phytochemicals interact with two of these enzyme key residues (Asp197 and Asp300), except α-pinene which forms interaction only with Asp300 amino acid. Overall, the natural metabolites studied in this work as well as acarbose were found to interact at the active site of α-amylase with similar amino acid residues as the native ligand did, especially the enzyme key residues.

Based on the findings, these identified compounds in the NS essential oil could be considered promising metabolites playing a major role in the inhibition of the human pancreatic α-amylase enzyme.

### 3.7 Molecular docking with human hemoglobin receptor

**FIGURE 7:**
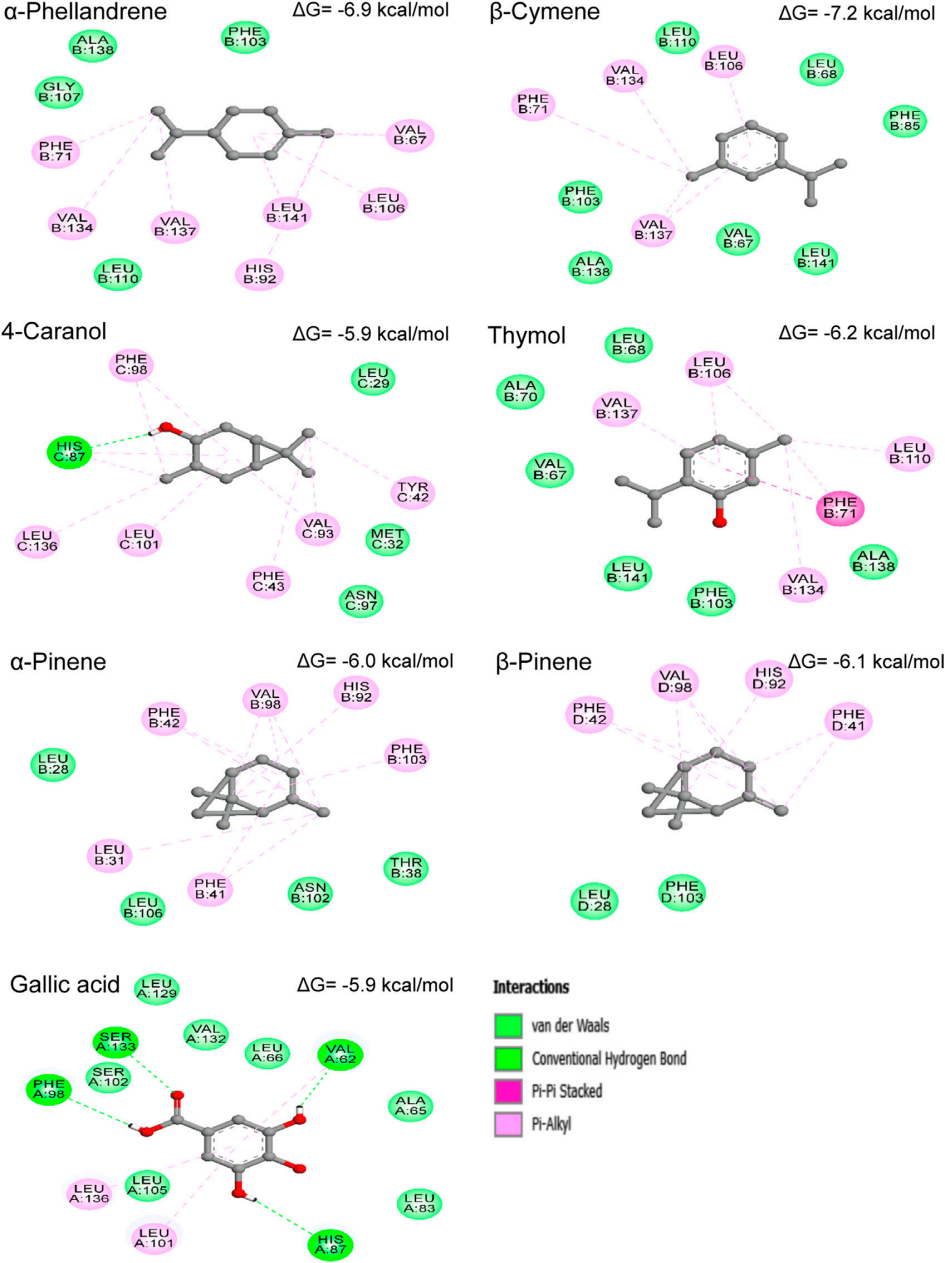
Binding energies (ΔG) and 2D-representations of the best-docked poses of the studied ligands in the active pocked of human hemoglobin.

To explore the putative binding mode of our compounds to the active site residues of human hemoglobin protein, molecular docking studies were performed. The results of the study have been depicted in Figure 7 in terms of binding energy (ΔG), involved receptor residues, and types of interactions.

As it can be seen in Figure 7, the binding forces of all selected compounds, except 4-caranol, were found to be least than that shown by standard drug (gallic acid). The binding energy of each molecule in order from highest to lowest i.e., gallic acid (−5.9 kcal/mol), 4-caranol (−5.9 kcal/mol), α-pinene (−6.0 kcal/mol), β-pinene (−6.1 kcal/mol), thymol (−6.2 kcal/mol), α-phellandrene (−6.9 kcal/mol), and β-cymene (−.2 kcal/mol). As observed in Figure 7, mainly hydrophobic interactions were responsible for the formation and the stabilization of compound-enzyme systems. Interestingly, the results reveal that the studied compounds bind to different chains of HbA, α-pinene, thymol, α-phellandrene, and β-cymene were found to interact with ‘B’ chain residues; β-pinene bind to ‘D’ chain; 4-caranol bind to ‘C’ chain; and gallic acid forms interactions with ‘A’ chain amino acids of HbA. In general, the molecules interacting with ‘B’ chain residues showed the best binding energy values.

The representation of the complex formed between gallic acid and HbA, indicating the presence of four hydrogen bonds with Val62, His87, Phe98, and Ser133; two Pi-Alkyl interactions with Leu101 and Leu136; in addition to seven VDW interactions with Leu105, Leu83, Leu129, Leu66, Ala65, Val132, and Ser102 residues. β-cymene -HbA complex was stabilized by Pi-Alkyl bonds with Leu106, Val134, Val137 and Phe71; and VDW forces involving the amino acids Phe103, Phe85, Leu68, Leu110, Leu141, Val67 and Val138. α-phellandrene showed similar interactions like β-cymene with slight modifications. Surprisingly, the two analog α-pinene and β-pinene were found to interact with the same residues (with more residues for α-pinene) despite the fact that they were bound to the different chains of HbA. The hydrogen atom of the hydroxyl group (OH) of 4-caranol forms a conventional hydrogen bond with His87, while the 6-ring and the methyl groups (CH_3_) form six Pi-Alkyl interactions. Moreover, the compound also binds with the three amino acids Leu29, Met32, and Asn97 *via* VDW forces. Thymol was surrounded by Leu106, Leu110, Val134, Val137, Phe71, Phe103, Val67, Leu68, Leu141, Ala70a, and Ala138 *via* Pi-Alkyl (with Leu106, Leu110, Val134, and Val137), Pi-Pi stacked (with Phe71) and VDW (with Phe103, Val67, Leu68, Leu141, Ala70, and Ala138) interactions.

## 4 Conclusion

The essential oil of *N. sativa* are endowed with an abundance of a variety of chemical compounds including α-Phellandrene, β-Cymene, and 4-caranol, among others. Our findings indicate that the volatile components of black cumin have a powerful ability to impede the pancreatic α-amylase and hemoglobin glycation. On the other hand, In order to determine the potential binding mode of the most prevalent chemicals found in NS essential oil, molecular docking studies were conducted, on human pancreatic α-amylase and human hemoglobin. The docking findings suggest these compounds as lead molecules against α-amylase and human hemoglobin. However, appropriate experiments using these phytochemicals should be performed to determine their *in vitro* and *in vivo* antidiabetic effects.

## Data Availability

The original contributions presented in the study are included in the article, further inquiries can be directed to the corresponding authors.
